# Wearable Tech for Health Monitoring of Environmental Events: Case Studies in Experimental Medicine

**DOI:** 10.1093/geroni/igaf122.1887

**Published:** 2025-12-31

**Authors:** Nelson Roque, Mindy Katz, Carol Derby

**Affiliations:** The Pennsylvania State University, University Park, Pennsylvania, United States; Albert Einstein College of Medicine, Bronx, New York, United States; Albert Einstein College of Medicine, Bronx, New York, United States

## Abstract

The COVID-19 pandemic underscored the need for adaptability in both daily life and scientific research. Using data from the Einstein Aging Study, we present case studies demonstrating the value of mobile and wearable technologies for behavioral monitoring in rapidly changing environmental conditions. First, we examine data availability during the COVID-19 pandemic, highlighting how smartphone-based assessments enabled real-time tracking of cognitive and psychosocial health measures—such as stress, affect, and social relationships—alongside the impact of different viral strains on cognition and behavior. Second, we explore the feasibility of low-cost wearable air quality monitors in capturing environmental exposures during two distinct events—Canada wildfires of March 2023 and holiday fireworks. Third, we examine how seasonal variations impact cognitive function measurement, highlighting the importance of considering temporal context in behavioral assessments. Our findings suggest that mobile monitoring methods offer a reliable approach to data collection in unpredictable and rapidly changing environmental conditions. Future research should identify the minimum data required for accurate outcome prediction, determining the threshold at which data remains meaningful.

